# Editorial: Psychological and Motor Associations in Sports Performance: A Mental Approach to Sports

**DOI:** 10.3389/fpsyg.2021.629944

**Published:** 2021-03-08

**Authors:** Donatella Di Corrado, Alessandro Quartiroli, Marinella Coco

**Affiliations:** ^1^Department of Human and Social Sciences, Kore University, Enna, Italy; ^2^Department of Psychology, University of Wisconsin–La Crosse, La Crosse, WI, United States; ^3^School of Sport, Health and Exercise Science, University of Portsmouth, Portsmouth, United Kingdom; ^4^Department of Biomedical and Biotechnological Sciences, University of Catania, Catania, Italy

**Keywords:** motor, sport, well-being, performance, sport psychology

## Introduction

“*Mens sana in corpore sano*” is used to paraphrase the idea that physical activity in all its forms is important and essential to foster and maintain mental and psychological well-being. The mind and the body must be considered as inseparable elements that represent two distinct natures of the same experience of living, communicating with and influencing each other. While they have been traditionally explored, analyzed, and treated separately, in recent years, the mind–body unit has witnessed a growing interest in the field of psychology and neuroscience applied to sports.

In this Research Topic, we have welcomed papers evaluating different approaches to sport performance. The articles of this collection examine in detail four main themes: (i) mental training to reach peak sports performance, (ii) emotional states associated with performance, (iii) the effects of personality traits on sports performance, and (iv) cognitive and psychophysiological characteristic associated with sports performance.

## Mental Training

Existing literature has widely highlighted the value of mental training in assisting participants in sports in developing and maintaining effective mental skills, which are important to reaching peak performance as well as to enjoy, and possibly succeed in, the competitive experience of sports. Six articles in this collection move from such a perspective. Mental toughness is considered a key factor of superior performance in a variety of domains including sport. Piggott et al. investigated whether skilled performance thrived across increased challenges in small-scale games in higher- and lower-skilled footballers. Mental toughness, decision-making, and motor skill execution were measured. Findings suggest higher levels of mental toughness may contribute to maintaining performance across the increased challenge of pressure within small-scale games. In a systematic review of the literature, *Neumann* explored the effects of attentional focus strategies during weightlifting tasks. The results have shown the advantages of an external attentional focus for motor skill learning. The use of music during training has been proven to have many health benefits. Patania et al. have assessed the relationship between the tempo of music and perception of effort during different metabolic demands. The results have shown that the beneficial effects of music are more visible in endurance training. Consequently, music can be an important tool to stimulate people engaging in low-intensity physical exercise. Pre-performance routines are essential skills prior to a competition for athletes. Using a qualitative research design, Yao et al. conducted in-depth interviews of 14 elite Chinese athletes in competitive diving and their coaches. The results showed that the divers' pre-performance routines encompassed four specific components. Emphasizing the key role of mental imagery, *Di Corrado et al*. have observed the cognitive abilities useful for mental imagery abilities, investigating modifications in mental imagery skills in competitive athletes and non-athletes. Competitive athletes showed higher scores on mental imagery skills than non-athletes. A final contribution is that of Zhang L. et al.. Investigating the neural efficiency hypothesis, these authors used functional magnetic resonance imaging to study the differences in brain activity between athletes imagining performing different movements. The results showed better temporal congruence between motor execution and motor imagery and vividness of motor imagery. Athletes were more effective in the representation of the motor sequences and the interoception of the motor sequences for their self-sport.

## Emotional States and Performance

Emotions play a central role in sports performance. Accordingly, it is important that athletes are able to draw on a range of strategies to improve emotional control. Eight contributions focused on this area of interest. Fernández et al. compared emotional intelligence and anxiety in combat sports of lower, intermediate, and higher-level female and male athletes. Results have shown that emotional intelligence is increased in high-level female athletes, while anxiety is prevalent in low-level female athletes. An athlete's emotional state may also affect the outcome of a competition by influencing performance both during training and while competing., Damonte et al. Sors, Lourido investigated the levels of sports burnout in 85 former agonist road cyclists, depending on whether their sport abandonment was relative or definitive. The results revealed that former agonist road cyclists still involved in cycling reported that they had experienced lower levels of emotional and physical exhaustion and sport devaluation during the last year practicing this specialty, with respect to both those who started practicing a different sport and those who definitively abandoned it. Poulus et al. explored the influence of mental toughness on stress and coping in 316 electronic sports athletes. Mental toughness was associated with the selection of more problem- and emotion-focused coping strategies and less avoidance coping strategies. Schüler and Wolff explored the impact of the presence or absence of achievement incentives on participants during a bicycle ergometer task. In a within-subject experimental design, they found that the lack of achievement incentives led to the worst performance, while the presence of these incentives did not lead to the best performance in participants with a strong achievement motive. Observing 309 karate competitions, the goal of the study conducted by Frigout et al. was to determine whether it would be possible to issue coaching requirements. The authors concluded that karatekas have to make decisions, such as when taking the risk to score points and penalties. Moreover, karatekas may decide to expose or protect themselves, create situations, or simply remain realistic, and adhere to the plan. Sport is an emotional experience not only for athletes. Sors, Lourido, Parisi et al. investigated how the crowd noise influences the decisions of basketball referees when examining videos of potential fouls, taking into consideration their level of competitive anxiety of referees (low vs. high anxiety). The results indicated that the decisions of referees with high anxiety might be more easily influenced by external factors like crowd noise. In a review of the literature, Guerrera et al. provided a general overview of the possible synergism between physical activity participation and the effects of antidepressants as part of a treatment for major depressive disorder. In conclusion, in an opinion article (Calleja-Gonzalez et al.) examined how different stressors impact wellness and performance due to constant experience of traveling often required in elite professional sport. For this reason, the authors indicated some key points in order to optimize performance, reducing the effect of traveling, and sleep disturbance.

## The Effects of Personality Traits on Sports Performance

Personality traits refer to individuals' characteristics that are stable over time. Three articles in this collection explore the role of personality traits in sport. Zhang G. et al. illustrated the relationship between the Big Five personality traits and self-control in 210 Chinese boxers and investigated self-efficacy as a mediator between the two variables. Results showed significant differences in self-control and self-efficacy among boxers of different competitive levels. Finally, self-efficacy mediated personality traits and self-control, indicating that personality traits predict self-control not only directly but also indirectly through self-efficacy. In another cross-sectional study, Baños et al. assessed how 890 high school students evaluate the professional personality competence of physical education teachers and its relation to students' satisfaction with school and satisfaction with life itself. The results showed that students' perceived competence, predicted self-determined satisfaction, which in turn corresponds to life satisfaction. In the last study, Valenzano et al. extended the work on individual differences in the relationship between personality traits and the cortisol response by examining the interaction effects of sex and the role category of 70 Italian adolescent elite dinghy sailors. Their findings showed positive associations among cortisol levels, extraversion, and consciousness in both male and female bowmen groups.

## Cognitive and Psycho-Physiological Characteristics

Over time, athletes show extraordinary skills in their favorite sport. While their sporting acumen may seem like a fundamentally physical attribute, it is actually supported by a range of cognitive skills spanning the sensorimotor line, from perception to action execution. Six contributions under this theme report on empirical studies. One such skill that has received considerable attention from experimental psychologists is the expert anticipatory advantage. Jalali et al. assessed the expert anticipatory advantage in 34 skilled tennis players via the approach “bubbles” (information is randomly removed from videos in each trial). Results showed that skilled tennis players could anticipate upcoming shots based on their opponent's body kinematics. Storniolo et al. have deepened the time delay between maximal sprint end and heart rate decay onset, exploring the relationship between sympathovagal balance and performance in 24 healthy adults. Results seem to confirm that a delay between sprint end and heart rate decay is a significant marker for the autonomic nervous system recovery after a sprint test. Papadopoulou et al. have observed the prevalence of relative age effect (RAE) in 72 selected and 53 non-selected youth female volleyball players. Their findings showed that RAE was not observed in the selected or non-selected players, (b) anthropometric and physiological characteristics did not differ among birth quarter groups, and (c) the relationship of anthropometric and physiological characteristics with age varied by performance group with stronger correlations observed in the non-selected than in the selected group. In a case study (28-year-old male), Dhawan demonstrated the effect of neuronal ensemble and memory formed during High-intensity aerobic training (VO2 max) and Target Heart-Rate (THR) training and the effect of reactivation of same memory on THR and performance. With this study, he showed how reactivation of previously acquired memory or using the stimulation from the neuronal ensemble of consolidated memory during a specific training event might exert similar physiological effects on exercise or the body to those that are learned during the memory acquisition phase. Focusing on cognitive features, Coco, Buscemi, Cavallari et al. determined whether aerobic exercise performed at two different intensities is able to affect executive functions in 20 healthy young male athletes. The results showed that a 30-min aerobic exercise is not associated with a worsening of executive functions as long as the blood lactate levels stay within the 4 mmol/l threshold. Finally, focusing on cognitive deficits, Coco, Buscemi, Perciavalle et al. explored whether the practice of scuba diving is capable of determining damage to the brain white matter (WM) in a dose-dependent manner and, consequently, a possible deficiency in their cognitive abilities in 54 professional scuba divers (35 men and 19 women). Their results lead to the conclusion that repeated dives, even performed in compliance with the current decompression tables, can progressively lead in the central nervous system to the formation of micro-lesions in the myelin sheet capable of altering the functioning of the neuron.

To conclude, we believe that all the studies in this Research Topic contributed to add support to the pluridimensional nature of motor and sport sciences; the findings, insights, and perspectives discussed in each paper can inspire new research and theory, opening up new horizons in this domain. We gratefully acknowledge the precious collaboration of the reviewers, editors, and staff of Frontiers.

## Author Contributions

All authors listed have made a substantial, direct and intellectual contribution to the work, and approved it for publication.

## Conflict of Interest

The authors declare that the research was conducted in the absence of any commercial or financial relationships that could be construed as a potential conflict of interest.

